# Carcinoma of the Uterus

**Published:** 1888-01

**Authors:** 


### *CARCINOMA OF THE UTERUS.*

German gynecologists have been busy with the statistics of the operation for the complete removal of the uterus for carcinoma. Material is now on hand to determine if the operation is a justifiable one. The testimony is to the effect that it is. It is, without doubt, superior to any other treatment of the cancerous uterus. Up to the close of the year 1886, 811 cases have been collected, with forty-seven deaths, or 15.1 per cent. With increased experience, the mortality is gradually decreasing, and we may expect it to continue to do so. As to immediate mortality, the operation shows better results now than the removal of the breast for cancer. The patients are usually prepared for the operation with an antiseptic vaginal injection. One stroke of the knife frequently suffices to open the pouch of Douglas, and display to view the posterior fornix, after which the cutting is done cautiously, paring with the finger-nail. Warm water is kept running over the surface, and no sponges are used. The peritoneum is opened on the one side, and one finger is passed in, which frees the broad ligament from the uterus. The ligament is then, in like manner, freed in the other side, up to which time the hemorrhage is very considerable. The bladder is then freed, which is done with the forceps and the knife.

The cut borders of the vagina are then united to the peritoneum, just as was done on the other side. It seems to be of little import whether the uterus is removed through an incision made posterior to the neck, or at the side or front of it. Some turn the uterus over; others draw it down and remove it. Some leave the opening in the floor of the pelvis; others close it. It may be drained either with iodoform gauze or a tube. If easily done, the tubes are also removed. It is not the custom here in Germany, as in France and England, to use the clamping forceps to restrain the hemorrhage from the ligamenta lata. The prognosis in the total extirpation of the uterus is quite as good as in the supra-vaginal operation, and is rapidly supplanting it.

